# Study of the Influence of the Wastewater Matrix in the Adsorption of Three Pharmaceuticals by Powdered Activated Carbon

**DOI:** 10.3390/molecules28052098

**Published:** 2023-02-23

**Authors:** Marina Gutiérrez, Paola Verlicchi, Dragana Mutavdžić Pavlović

**Affiliations:** 1 Department of Engineering, University of Ferrara, Via Saragat 1, 44122 Ferrara, Italy; 2 Department of Analytical Chemistry, Faculty of Chemical Engineering and Technology, University of Zagreb, Trg Marka Marulića 19, 10000 Zagreb, Croatia

**Keywords:** adsorption, diclofenac, sulfamethoxazole, trimethoprim, dissolved organic matter, powdered activated carbon, wastewater

## Abstract

The use of powdered activated carbon (PAC) as an absorbent has become a promising option to upgrade wastewater treatment plants (WWTPs) that were not designed to remove pharmaceuticals. However, PAC adsorption mechanisms are not yet fully understood, especially with regard to the nature of the wastewater. In this study, we tested the adsorption of three pharmaceuticals, namely diclofenac, sulfamethoxazole and trimethoprim, onto PAC under four different water matrices: ultra-pure water, humic acid solution, effluent and mixed liquor from a real WWTP. The adsorption affinity was defined primarily by the pharmaceutical physicochemical properties (charge and hydrophobicity), with better results obtained for trimethoprim, followed by diclofenac and sulfamethoxazole. In ultra-pure water, the results show that all pharmaceuticals followed pseudo-second order kinetics, and they were limited by a boundary layer effect on the surface of the adsorbent. Depending on the water matrix and compound, the PAC capacity and the adsorption process varied accordingly. The higher adsorption capacity was observed for diclofenac and sulfamethoxazole in humic acid solution (Langmuir isotherm, *R*^2^ > 0.98), whereas better results were obtained for trimethoprim in the WWTP effluent. Adsorption in mixed liquor (Freundlich isotherm, *R*^2^ > 0.94) was limited, presumably due to its complex nature and the presence of suspended solids.

## 1. Introduction

Pharmaceuticals are one of the most common organic micropollutants found in wastewater. Among pharmaceuticals, nonsteroidal anti-inflammatory drugs (NSAIDs) and antibiotics are in the spotlight due to their high consumption and/or recalcitrant nature [1,2]. In wastewater treatment plants (WWTPs), the core treatment is biological degradation, and even though some pharmaceuticals are highly biodegradable, the concentrations found in WWTP effluent are still an issue, because WWTPs are not designed to remove them [3]. In this way, advanced treatments have gained interest and have been gradually implemented over the last few years [4,5,6]. These treatments include activated carbon adsorption (in powder or granules), which offers the advantage of being able to remove a wide range of compounds. This is particularly relevant in wastewater treatment, where organic micropollutants often occur as a “cocktail”, and tens to hundreds of substances can be found at the same time [7]. Indeed, the removal of many recalcitrant substances relies almost uniquely on sorption processes [8]. Powdered activated carbon (PAC) is known for being a very flexible option that can be added to existing treatment lines (i.e., addition to the biological tank) or as a polishing treatment to treat the secondary effluent (i.e., in a new contact tank) [9,10]. PAC is used to enhance the removal of substances via adsorption and to promote diverse removal mechanisms with the main aim of obtaining synergistic effects (such as enhanced biodegradation).

Adsorption onto activated carbon, which is driven by the properties of the adsorbent and absorbate as well as the water quality, is a complex process that is not fully understood [11]. When considering the application of PAC in WWTPs, the potential enhancement of the removal of pharmaceuticals depends on many factors for which the extent of their influence is challenging to consider altogether [12]. Activated carbon is a porous adsorbent of which the adsorption capacity depends on its surface properties (specific surface area, pore volume, functional chemical groups) [13]. Pharmaceuticals instead depend on their physicochemical characteristics (compound charge, hydrophobicity, molecular weight, etc.) to be adsorbed, which usually leads to competition effects such that some substances tend to adsorb more easily than others. Moreover, the overall adsorption process depends also on the conditions in which it occurs, such as the water matrix. The constituents of the water matrix and, more specifically, the dissolved organic matter (DOM), may influence the adsorption process. DOM is formed by many fractions that differ in size (building blocks, biopolymers, humic acids, low molecular weight organics, etc.), which may limit the adsorption of pharmaceuticals by blocking the pores on the PAC surface or by direct competition for the adsorption sites [14,15]. Pharmaceuticals may also interact with the DOM present in the liquid phase or the DOM that is adsorbed onto the PAC surface. The results of the interaction may enhance or diminish the adsorption onto PAC, depending on the tested compounds and conditions [11,16,17]. In our previous paper [18], the removal efficiencies of a vast selection of organic pollutants at trace levels were compared and discussed in different MBR coupled to PAC treatment configurations. Specifically, the PAC was added either inside the biological tank of the bioreactor (mixed liquor) or in a post-treatment unit to treat the MBR permeate. Results indicated that the effect of the PAC dosage point was dependent on the compound under study. In general, the presence of suspended solids and the complex nature of the mixed liquor requires higher doses of PAC compared to the MBR permeate to achieve equivalent removal efficiencies [19]. Due to the presence of the micro- or ultra-filtration membranes in the bioreactor, the MBR permeate is free of suspended solids [20]. In light of the foregoing information, the use of synthetic water matrices (i.e., humic acid solution) can act as a means to understand the adsorption process under certain DOM constituents [17].

**Figure 1:**
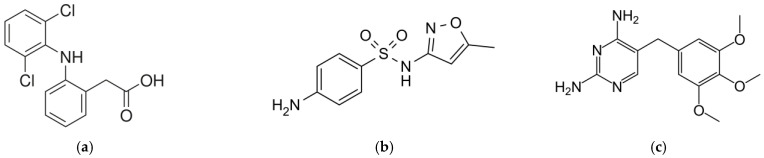
Molecular structure of (**a**) diclofenac, (**b**) sulfamethoxazole and (**c**) trimethoprim.

Because the adsorption onto PAC is influenced by the adsorbate’s properties, three pharmaceuticals (Figure 1), namely diclofenac (DCF), sulfamethoxazole (SMX) and trimethoprim (TMP), were selected. These compounds have been subjected to several studies due to their low-to-moderate removal in WWTPs and the potentially harmful effects on the environment that they may entail [21,22]. Additionally, they differ in hydrophobicity (octanol–water partition coefficient, *K*_ow_) and charge at the pH of the wastewater. These parameters are commonly used to predict the effectiveness of the addition of PAC on the wastewater treatment line [23].

DCF is a non-steroidal anti-inflammatory drug (NSAID) used to treat pain and inflammatory disorders. Banned in many countries of Southeast Asia [24], DCF was selected for the first Watch List (Decision 2015/495) for Union-wide monitoring in Europe [25]. DCF is a weak electrolyte (Figure 1) with high hydrophobicity (log*K*_ow_ = 4.3) [26] that predominates in its anionic form in wastewater [27]. Compared to other NSAIDs, DCF shows inefficient and variable removal efficiencies in WWTPs, with great discrepancy among the literature data [28]. In this way, the addition of PAC has been shown to be beneficial, albeit the removal efficiencies found in the literature still show great variability (32–99%) [18].

SMX is a bacteriostatic antibiotic commonly prescribed in combination with TMP. SMX is an anionic compound with very low hydrophobicity (log*K*_ow_ = 0.8) [26]. Although these chemical properties are disadvantageous for the direct adsorption of SMX onto PAC, it has been shown that the addition of this adsorbent to the biological tank of a membrane bioreactor (MBR) may increase the removal of this compound [29]. Moreover, batch adsorption isotherms obtained by Li et al. [8] estimated a maximum adsorption of (*q*_m_) 0.017 mg/g.

TMP is an antibiotic that was included in the European Watch List in 2020 (Decision EU 2020/1161) and was maintained in the recent update published in 2022 (Decision 2022/1307) [30,31], for which its monitoring and related research are promoted. It is a relatively hydrophilic compound with a low tendency for sorption onto the sludge of the WWTPs [21]. It has been generally classified as moderately removed in WWTPs, with better removal efficiencies when PAC is added inside the bioreactor compared to when it is added as a post-treatment [18].

Adsorption batch experiments and mathematical models can be useful tools to examine the conditions under which PAC adsorption takes place and to predict adsorbent response to such conditions [32]. In previous research, the application of adsorption models has been of great value to understand the mechanisms of adsorption of certain pollutants on porous adsorbents such as PAC [33]. However, only a few studies have applied these models to study the effect of varying concentrations of DOC [6] and DOM constituents [15,16,17] in the adsorption of pharmaceuticals in wastewater. Indeed, the potential positive effect of these interactions between DOM and pharmaceuticals has been rarely documented and quantified [11,16]. With regard to the adsorbates, the influence of their physicochemical properties (polarity, charge and hydrophobicity) in adsorption has been the subject of study in the literature [6], but rarely has the literature focused on the subsequent potential competition effect caused by their different affinity towards PAC under realistic conditions of wastewater treatment [34].

For all the above-mentioned reasons, the adsorption of three pharmaceuticals onto PAC is investigated under different conditions using four different approaches. First, the adsorption capacity of PAC for the three target compounds is determined experimentally, and the adsorption process is described by three isotherm models (Linear, Langmuir and Freundlich) and three kinetic models (Lagergren’s pseudo-first-order, pseudo-second-order and intraparticle diffusion model (IPD)). Second, the potential competition effect among pharmaceuticals due to their different physicochemical properties (charge, hydrophobicity) is evaluated. Third, the potential influence of the water matrix is assessed by comparing the adsorption process (kinetics, isotherms, experimental adsorption capacity) in ultra-pure water, humic acid solution, permeate of a full-scale membrane bioreactor (MBR) and mixed liquor from the nitrification tank of the same MBR. Finally, the interaction between the pharmaceuticals and the DOM on the adsorption onto PAC is studied.

## 2. Results and Discussion

### 2.1. Effect of the Contact Time and Initial Concentration of Pharmaceuticals

**Figure 2:**
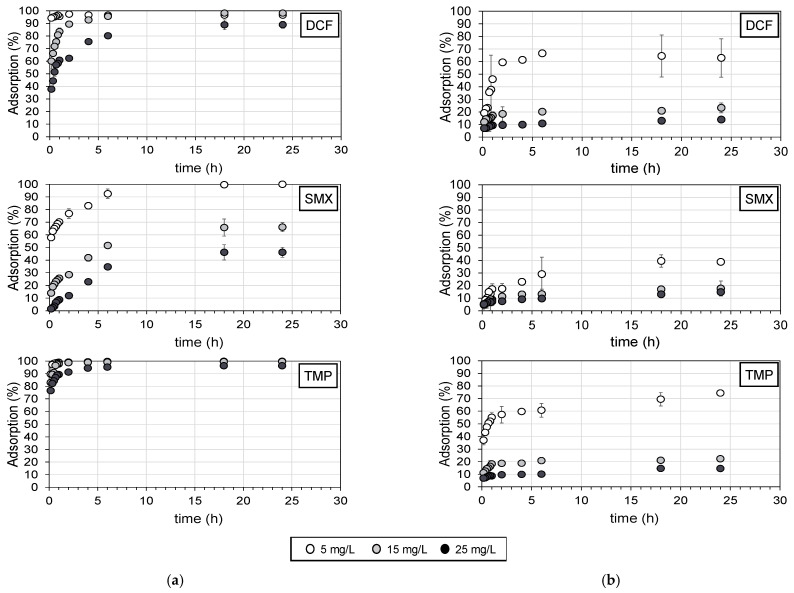
Kinetics of adsorption of DCF, SMX and TMP at three different concentrations in Milli-Q water with (**a**) 1 g/L of PAC and (**b**) 0.1 g/L of PAC at different contact times (10 min–24 h). Error bars indicate the standard deviation.

In order to determine the time needed to reach the maximum adsorption of the target pharmaceuticals onto PAC, adsorption experiments at various contact times were conducted. For this purpose, individual solutions of each pharmaceutical were tested at three concentrations (5, 15 and 25 mg/L) with two concentrations of PAC (0.1 and 1 g/L) at various contact times (10, 20, 30, 40 and 50 min and 1, 2, 4, 6, 12, 18 and 24 h). Figure 2 shows the removal (in terms of % of adsorption) of the three target compounds over time (10 min–24 h) in Milli-Q water with 1 and 0.1 g/L of PAC. All target compounds reached the equilibrium within 24 h, with very little difference in the adsorption between 18 h and 24 h, indicating that no more molecules could be adsorbed. In this way, 24 h was taken as the equilibrium time for the adsorption isotherms.

TMP was almost completely removed by the adsorption onto PAC (1 g/L) at 24 h (96–99.8%), followed by DCF (88–97%) and SMX (46–99.9%). TMP was the compound with the fastest kinetics, with removal from 77% (for the initial concentration of 25 mg/L) to 90% (for the initial concentration of 5 mg/L) in the first 10 min of agitation. SMX instead was the compound with the lowest rates and overall adsorption, depending on the initial concentration. In the first 10 min, 57% of the compound was adsorbed for 5 mg/L (maximum adsorption of 99.9% after 24 h), whereas only 1.5% was adsorbed for 25 mg/L (at 24 h, only 46% of the compound was adsorbed).

Lower adsorption percentages were found when PAC was added at 0.1 g/L for all OMPs in all tested shaking times (Figure 2). At an initial concentration of 5 mg/L, adsorption of 39%, 63% and 74% was obtained at 24 h for SMX, DCF and TMP, respectively. On the other hand, maximum adsorption of approximately 15% was obtained for all OMPs at 25 mg/L. From Figure 2, it can be seen that the adsorption rate was particularly high within the first ten minutes in all tested OMPs with an initial concentration of 15 and 25 mg/L. The adsorption percentage that was reached in 10 min was approximately 50% of the total adsorption that was obtained after 24 h. As an example, the adsorption of DCF at 10 min was 7%, and after 24 h, it was 15% (Figure 2b). After the first ten minutes, the rate of adsorption was considerably low until it reached equilibrium.

Note that adsorption seems to be dependent on the initial concentration of the pharmaceuticals (Figure 2). Higher adsorptions were found at the initial concentration of 5 mg/L compared to 15 and 25 mg/L for DCF, SMX and TMP, indicating that the adsorption of pharmaceuticals onto activated carbon is dependent on their initial concentration.

### 2.2. Kinetics

Sorption of the tested pharmaceuticals has proved to be a fast process overall. However, the behavior of each compound was different, presumably due to their physicochemical properties and the initial conditions of the experiments (i.e., the concentrations of the adsorbent and the adsorbate).

The kinetics models were applied to all the tested concentrations of pharmaceuticals and PAC, even though the behavior should be the same regardless of the initial concentration ratios. In this way, a vast data set was covered, and the reliability of the results obtained was assured. The kinetics followed a pseudo-second-order model for the three target compounds at the two tested PAC concentrations (1 and 0.1 g/L). The sorption rate constants (*k*_1_ and *k*_2_), *q*_e, calc._, *q*_e, exp_. and correlation coefficients (*R*^2^) are shown in Table 1. The correlation coefficients of the adjustments were very close to the unity (*R*^2^ > 0.98), with no significant differences between the experimental *q*_e_ (*q*_e, exp._) and calculated values (*q*_e, calc._), suggesting that the sorption is governed by the number of available active sites [34,35]. The lowest *q*_e, exp._ values were obtained via SMX in all tested concentrations. The maximum amounts of adsorbed pharmaceuticals onto PAC (*q*_e, exp._) were the highest at the lowest PAC concentration and vice versa. The values obtained were in the range of 4826–24,083 µg/g for 1 g/L of PAC and 19,398–37,184 µg/g for 0.1 g/L of PAC based on the three tested OMPs. Furthermore, higher initial concentrations (*C*_0_) of tested pharmaceuticals led to higher values of *q*_e, exp_. The results indicate that PAC adsorption capacity in the equilibrium increases when it is found at low concentrations with high concentrations of the absorbate (i.e., pharmaceutical) in the solution.

As anticipated in Figure 2, the fastest kinetics (*k*_2_) were obtained with the lowest pharmaceutical concentration (5 mg/L) for all the tested compounds except for TMP at 1 g/L PAC. Depending on the initial concentration, *k*_2_ changes by at least one order of magnitude, indicating that the initial OMP concentration seems to have a significant role in the sorption kinetics.

In parallel with pseudo-first and second-order models, the data were fit into the IPD. Previous studies have reported that the removal of pharmaceuticals via adsorption onto PAC does not fit IPD because the rate of adsorption is controlled by one or more stages [34,36,37]. Nevertheless, although the model does not fit, it is known that in porous adsorbents such as PAC, intraparticle diffusion plays a major role in the adsorption process [36]. The IPD model may be useful for predicting the reaction pathways and the rate-controlling step in the transport from the water matrix to the active sites [38]. For porous adsorbents such as PAC, the adsorption process is differentiated into four stages, as stated originally by Walter and Weber [39]. The first stage is the transfer of the target pollutant to the solution (bulk transport); the second is the film diffusion, in which the adsorbate is transported from the bulk phase to the external surface of the PAC; the third stage is the diffusion of the adsorbate molecules along the adsorbent surface or through the pores (i.e., intraparticle diffusion), which is defined as the rate-controlling step in the IPD model; and the fourth stage is when the adsorption bond is formed between the OMP and the active site. When the adsorption onto PAC is controlled via intraparticle diffusion, stages 1, 2 and 4 occur very quickly, and the intraparticle diffusion is the only rate-controlling step. As a result, the IPD model adjustment should show a linear relationship between *t*^1/2^ and *q*_t_ with a null intercept (*C* = 0). In the original linear form of the IPD [40], only the second, third and fourth stages are considered because bulk transport does not directly relate to the solid–liquid sorption process.

In this study, the *q*_t_ versus *t*^1/2^ plot showed multi-linearity with three different slopes, indicating that the adsorption process is governed by a multistep mechanism, which is differentiated via the three abovementioned stages [38]. The fitting data for the model are shown in Table 2. First of all, it can be seen that the values of the rate constant (*k*_id_) follow the following order: *k*_id1_ > *k*_id2_ > *k*_id3_, for all the samples tested. *k*_id_ values are also at a higher *C*_0_. The fact that the third stage is the lowest is due to it corresponding to the equilibrium state in which intraparticle diffusion gradually slows down; the OMPs come into contact with the active sites, and the final equilibrium is reached, resulting in the corresponding plots being nearly horizontal lines [41,42]. Regarding constant *C*, the results show that C ≠ 0 in all samples tested, and increasing values from *C*_1_ to *C*_3_ were found for DCF and TMP. Constant *C* is associated with the thickness of the boundary layer, which implies that there is a higher boundary layer effect within the pores (and active sites) of the activated carbon compared to the outer surface. According to Rudzinski and Plazinski [43], negative values of intercept *C* observed for SMX can be explained by the presence of a “subsurface” region close to the surface of PAC on which the concentration of the adsorbate is different from that in the bulk phase, which affects the rate of the surface reactions (pseudo-second-order kinetics) at the initial times.

Although the adsorption onto PAC is governed via a multi-step mechanism, and intraparticle diffusion is not the only rate-limiting stage in the adsorption process, the IPD model was useful for understanding the sorption mechanisms of the three target pharmaceuticals. In general, it can be deduced that once the compound passes through the boundary layer from the bulk phase to the external surface of the PAC, it slowly moves from the macropores to the active sites, decreasing the adsorption rate. The adsorption also seems to be determined by a boundary layer effect that increases its relevance in the latter stages of the adsorption process.

### 2.3. Sorption Isotherms in Ultra-Pure Water and Competition Effect

Pharmaceutical concentrations tested for isotherm determination were in the range of 5–25 mg/L, whereas PAC concentration was between 0.1 and 1 g/L. The equilibrium time was set at 24 h. PAC concentrations were selected in accordance with the literature [8,29,44]. The pharmaceutical concentrations were the lowest allowed by the analytical method. Due to the high adsorption capacity of the PAC, lower concentrations would be almost completely adsorbed and would not be detectable. The sorption coefficient of the linear sorption, together with the sorption parameters derived from the Langmuir and Freundlich models, and regression coefficients (*R*^2^) are listed in (Table 3, individual solutions). From the analysis of the results obtained, it emerges that regression coefficients for linear sorption (0.783–0.96) were significantly lower than the Langmuir and Freundlich models (*p* < 0.05) for all three tested compounds, which means that the model does not fit the adsorption data very well. On the other hand, no significant differences were found between Langmuir and Freundlich for DCF and TMP, whereas the Freundlich model provided better *R*^2^ coefficients for SMX. This finding is in agreement with previous studies in the literature [36,37,45], where very similar *R*^2^ values were obtained, and no statistical analyses were performed to determine the best-fitting equation. Langmuir and Freundlich isotherms are the most used for describing the adsorption of porous adsorbents in wastewater, but further investigations on isotherm modelling may be needed to best describe the adsorption process.

Considering *K*_d_, *q*_m_ and *K*_F_ parameters, the results observed in the kinetic studies were confirmed once again, and the pharmaceuticals that were better adsorbed in PAC are as follows: TMP, DCF and SMX. On the other hand, the term 1/n of Freundlich isotherm represents the intensity of adsorption. Because the values found for all compounds are less than 1, it can be assumed that there is a good affinity between the adsorbates and the adsorbent and that chemical adsorption occurs.

Complex mixtures of pharmaceuticals are usually found in urban wastewater [7]. The diversity of the nature and target use of these substances is usually reflected in their physicochemical properties (e.g., hydrophobicity, solubility, charge, molecular weight). When PAC is applied for the removal of pollutants in wastewater, adsorption depends on the interactions between the compound and the adsorbent surface, and the aforesaid pharmaceutical properties may be the key to understanding and predicting the adsorption tendency of the compound. For these reasons, it is of great importance to understand the competitive effect among pharmaceuticals when considering adsorption onto activated carbon. The target compounds are expected to be adsorbed to varying degrees, and the competition for the adsorption sites may vary depending on the initial concentration and physicochemical properties of the compound.

To evaluate the competitive effect of DCF, SMX and TMP, the results of adsorption isotherms of the mixture (Table 3) and kinetic studies (Table 4) are presented. As for individual solutions, no statistical differences among isotherm models were found, except for the significantly lower *R*^2^ of linear isotherm in the case of DCF (*p* < 0.05). Despite the lack of significance, the regression coefficients for the Langmuir isotherm are slightly higher, indicating that monolayer adsorption on the PAC surface is assumed and that the differences in adsorption among pharmaceuticals depend on the affinity of the compound to the PAC surface. Although there were no differences between the maximum adsorption capacity (*q*_m_) among the pharmaceuticals, the Langmuir adsorption constants (*K*_L_) were significantly lower for SMX (*p* = 0.018). Similarly, *K*_d_ and *K*_F_ showed significant differences among tested compounds (*p* < 0.05), with higher coefficient values in the following order: TMP > DCF > SMX.

When comparing isotherm coefficients between individual solutions and the mixture, only *K*_F_ and *K*_d_ were found to be significantly lower in the mixture compared to the individual solution in SMX. In this sense, although no significant differences were found for the other parameters (*q*_m_, *K*_L_) and compounds (DCF, TMP), higher values were found in the individual solutions, indicating that there is some competition effect, especially for SMX.

Kinetics studies were used to evaluate whether the rate and mechanism of adsorption of each compound in the mixture (Table 4) varied in comparison with individual solutions (Table 1). In this regard, the same experimental conditions were applied to compare the results with accuracy. In the mixture, the results show that the compounds followed a pseudo-second order equation (Table 4), with no significant differences between *q*_e,exp_ and *q*_e,calc_ (*p* > 0.05). Despite there being no differences between the kinetic coefficients (*k*_2_) for the individual solutions and the mixture, the *q*_e,exp_ values were overall greater in the individual solutions compared to the mixture (*p* = 0.01). Indeed, considering the removal of the compounds in the liquid phase, removal efficiencies were found to be between 23% and 27% higher in the individual solutions at 5 mg/L of the three tested compounds compared to the mixture (e.g., 62.9% versus 36.9% for DCF).

**Figure 3:**
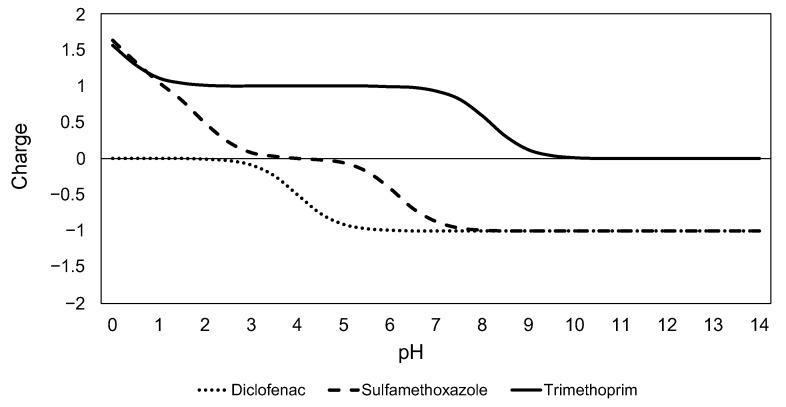
Changes in the ionization state of DCF, SMX and TMP as a function of the pH. J Chem for Office (20.11.0, ChemAxon, https://www.chemaxon.com, accessed on 11 June 2021) was used for calculating the ionization state.

In general, TMP was the compound that adsorbed best at PAC. TMP is the only tested pharmaceutical that is found mainly in its cationic form at the pH of water and wastewater (pH 6–8) (Figure 3). Regardless of their other physicochemical properties, cationic compounds are proven to be well removed on PAC hybrid systems, due to the electrostatic interactions with the negatively charged surface of most manufactured PACs [5,6]. The charge of ionizable compounds is the conducting parameter that determines their adsorption onto PAC [12]. In water and wastewater, DCF and SMX are present mainly in their anionic form, and the expected removal via PAC is lower. In the absence of positive electrostatic interactions, hydrophobicity (measured log*K*_ow_) becomes the critical factor for predicting adsorption. SMX is an anionic compound with very low hydrophobicity (log*K*_ow_ = 0.79) compared to that of DCF (log*K*_ow_ = 4.26). Both properties are responsible for the lower adsorption of SMX onto PAC in the tested conditions.

### 2.4. Influence of the Water Matrix

In wastewater treatment, the water matrix influences the adsorption process as well as the physicochemical properties of the of the adsorbates. In hybrid systems combining biological treatment with adsorption, PAC can be added in the biological tank (in contact with the mixed liquor) or as a polishing treatment for the secondary effluent [9,32]. Because the constituents and quality of the wastewater change along with the treatment step, it is essential to study the influence of the water matrix on the adsorption of contaminants. One of the most important parameters to consider is the presence of dissolved organic matter (DOM) [46]. DOM is constituted of fractions of different sizes (i.e., building blocks, humic and fulvic acids, biopolymers and low molecular weight organics) which may interfere with the adsorption to varying degrees [15] by blocking the PAC pores or competing with the pollutants of interest for adsorption sites. Indeed, the addition of fresh PAC is required to maintain high removal efficiencies, because the PAC surface becomes saturated over time mainly due to the adsorption of the DOM present in the wastewater [9,46]. In addition, the effect of PAC saturation is more pronounced for anionic compounds, because DOM is negatively charged at the overall pH of wastewater and interferes with the adsorption of anionic compounds through electrostatic repulsion [6]. However, the effect of the presence of DOM is still unclear. Many studies report that DOM has no significant effect or may even have a positive effect on the adsorption of some pharmaceuticals, depending on the experimental conditions [11,14,47].

The influence on the water matrix was studied by performing adsorption batch experiments in ultra-pure water, humic acid (HA) solution, MBR permeate and mixed liquor and comparing the obtained experimental results and isotherm modelling. Although the composition of DOM in the MBR permeate and the mixed liquor was not determined, the total DOC concentration was measured for the HA solution (29.35 mg/L), MBR permeate (4.1 mg/L) and mixed liquor (4.7 mg/L). It should be noted that the DOC concentration in the MBR permeate and that in the mixed liquor are quite similar, despite their different nature. Mixed liquor possesses a high concentration of total suspended solids (6 g/L) compared to MBR permeate (5.4 mg/L). In this case, the solid phase mixed liquor was included in the adsorption experiments, because it can act as an adsorbent and influence the interactions between pharmaceuticals and PAC.

**Figure 4:**
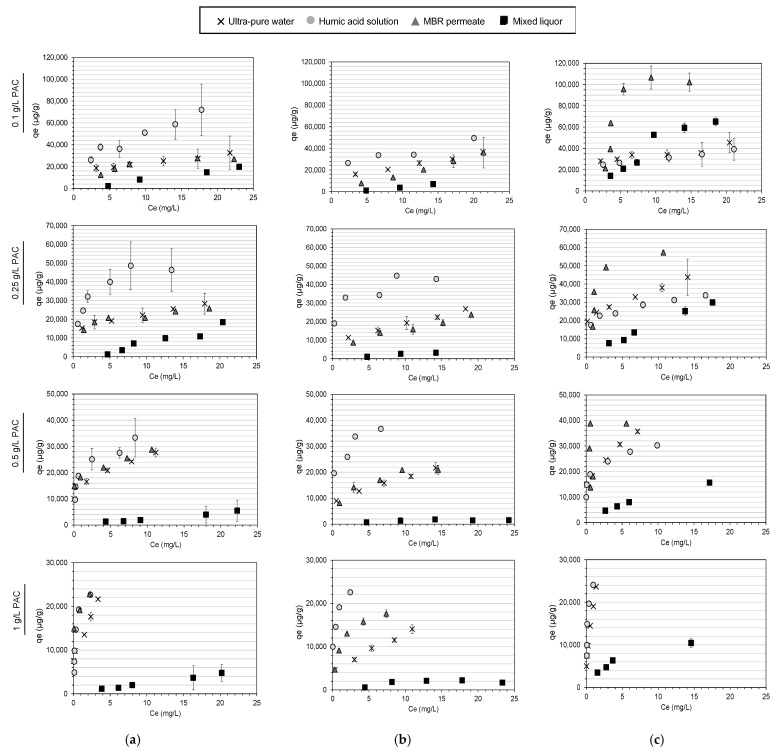
Experimental equilibrium adsorption capacity of (**a**) DCF, (**b**) SMX and (**c**) TMP at four different PAC concentrations (0.1, 0.25, 0.5 and 1 g/L) in ultra-pure water (×), humic acid solution (○), MBR permeate (▲) and mixed liquor from a WWTP (■). Error bars indicate the standard deviation.

Experimental equilibrium adsorption capacities of DCF, SMX and TMP for each water matrix are depicted in Figure 4. Sorption parameters from isotherm models and regression coefficients for each water matrix are listed in Table 5.

The adsorption mechanisms and, therefore, the isotherm models that describe them may vary from compound to compound, as described in the literature [48]. Similarly, they appear to depend on the water matrix in which adsorption occurs. As mentioned earlier, both the Langmuir and the Freundlich models fitted the results of DCF and TMP in ultra-pure water very well, whereas for SMX, the Freundlich model provided a better fit. Nonetheless, the regression coefficients of the Langmuir model for SMX are very high (*R*^2^ > 0.956). As for ultra-pure water, both Langmuir and Freundlich isotherms had very similar regression coefficients in MBR permeate, and there was not a model that fitted the results better for any of the compounds tested. None of the Langmuir parameters (*K*_L_ and *q*_m_) differed significantly between the pharmaceuticals. Instead, the Langmuir isotherm clearly fitted the *q*_e_ versus *C*_e_ plot in the humic acid solution, whereas the Freundlich isotherm had significantly higher *R*^2^ values in the mixed liquor. In the Langmuir isotherm, monolayer adsorption onto the PAC surface is assumed with a fixed number of energetically equivalent sites, whereas the Freundlich isotherm is considered to be an empirical expression for multilayer adsorption with different energy in the active sites [35]. Mixed liquor is expected to represent a much more complex matrix because it was extracted from the biological reactor, where most of the biological and chemical transformations take place for the removal of contaminants. In previous studies, it has been observed that given similar DOC-pharmaceutical concentrations, DOM composition may induce a stronger adsorption competition effect depending on the type of water (i.e., drinking water compared to WWTP effluent) [14]. In this way, the results are not surprising and confirm that adsorption mechanisms change depending on experimental conditions.

Assuming that the Freundlich isotherm had the best fit for all the water matrices, the higher average *K*_F_ values were found as follows: HA solution, ultra-pure water, MBR permeate and mixed liquor. Higher *K*_F_ values correspond to a higher adsorption capacity of the PAC (*q*_e_) for the same equilibrium concentration (*C*_e_) for all three compounds. As shown in Figure 4, higher PAC loads were obtained in the humic acid solution for DCF and SMX, followed by ultra-pure water and MBR permeate, with very similar results (*p* > 0.05). On the other hand, PAC loads were found to be the lowest in the mixed liquor for all pharmaceuticals. For TMP instead, the best results were obtained in the MBR permeate, followed by ultra-pure water, humic acid solution and mixed liquor. Indeed, for 1 g/L of PAC, the remaining concentrations of TMP in the MBR permeate were too low to perform the isotherm modelling. For 0.1 g/L of PAC, an unexpected increase in the adsorption capacity was achieved at higher TMP concentrations in the mixed liquor, not following the trend in the other PAC concentrations. Although the overall results are not consistent with other studies [11,49], in which the adsorption capacity in wastewater was systematically lower compared to that in ultra-pure water, it is possible that positive interactions between the humic acids and MBR effluent DOM lead to an increased adsorption capacity of PAC. Moreover, in real wastewater systems, DOM is present at a concentration of three to six orders of magnitude higher than organic micropollutants (mg/L compared to µg/L—ng/L). In our experimentation, the extent of the effect of DOM may be limited or altered because the *C*_0_ of the tested pharmaceuticals ranged from 5 to 25 mg/L. In all water matrices, the highest PAC loadings (*q*_e_) were observed at the lowest PAC concentration (0.1 g/L) and maximum pharmaceutical concentration (25 mg/L) for all the water matrixes and compounds (Figure 4).

It has been observed that the adsorption of some pharmaceuticals is promoted by the presence of humic acid in soils and sediments, suggesting that the presence of these substances may positively influence the sorption affinity for the adsorbent. Humic substances, which are also commonly found in wastewater, are known to act as carriers of organic micropollutants such as pharmaceuticals [50]. Due to their mobility and ability to form complexes with organic and inorganic species, commercial HAs may contain trace elements (e.g., ions, heavy metals) that contribute to the adsorption of further organic compounds (i.e., diclofenac) in adsorption experiments [50]. In another study, the formation of ciprofloxacin–HA complexes has been reported as a “false positive adsorption” when testing the sorption capacity of various adsorbents [17]. According to Behera et al. [33], the pharmaceutical–HA complex would be able to adsorb onto the surface of the adsorbent. These authors also suggest that the free pharmaceuticals in the solution could adsorb onto the already adsorbed HA, leading to an increase in adsorption [17]. On the other hand, the high concentrations of HAs in our study (29.35 mg/L) may enhance the sorption of some pharmaceuticals via hydrophobicity. Even if the interaction between DOM and pharmaceuticals is not expected, the presence of HAs may promote the adsorption through the PAC in the solution. The adsorption of dissolved humic substances has been proved to reduce the aggregation of carbon nanotubes, thus increasing the surface area available for adsorption by two orders of magnitude, increasing the change in the hydrophobic interactions between the adsorbent and SMX [47]. This could explain the increased adsorption of DCF and SMX, two anionic compounds for which the electrostatic interactions with the DOM would not be primarily considered. For the aforementioned reasons, the increased adsorption capacity of PAC in the HA solution is not surprising. Although there is no single phenomenon that explains the observed results, the literature data confirm that the presence of humic substances can affect the adsorption of organic compounds such as pharmaceuticals in several ways.

In the case of the MBR permeate, the results show that the presence of DOM had no negative effect on drug adsorption, with no statistical differences from ultra-pure water for DCF and SMX (*p* > 0.05) and with an increase in the adsorption capacity of PAC for TMP (*p* < 0.05). Because the concentration of the pharmaceutical influences the experimental adsorption values (with the highest *q*_e_ values at *C*_0_ of 25 mg/L in all water matrices), it may be that DOC is not high enough in the solution to cause a decrease in adsorption compared with ultra-pure water. In any case, the results show that the adsorption of TMP in the MBR permeate was enhanced, probably due to the above-mentioned reasons related to HAs and, in particular, to the fact that TMP is positively charged, which could favor the interactions with negatively charged DOM. PAC added to the secondary effluent of full-scale WWTPs has been proved to provide a better quality effluent (i.e., lower TMP concentration) compared to PAC added in the biological reactor, in contact with the mixed liquor, indicating that the DOM constituents of the MBR permeate have a different effect on the adsorption of TMP onto PAC [18]. Indeed, TMP was not the only compound with lower adsorption in the mixed liquor (Figure 4). Even with the very similar DOC concentration, the differences in the adsorption capacity of PAC between the MBR permeate and mixed liquor indicate that the DOM constituents play a significant role in the adsorption process. Although HAs appeared to favor adsorption, low molecular weight organics have been demonstrated to limit the process due to direct competition for the adsorption sites [35]. However, it should be noted that the experiments conducted aimed to reproduce the adsorption process under real WWTP conditions, and, therefore, the solid fraction of the mixed liquor was included in the adsorption batch experiments. Because some pharmaceuticals are also able to adsorb onto the sludge [27], additional adsorption experiments were performed without the addition of PAC to quantify the adsorption onto the solid phase of the mixed liquor (dried sludge). The results of the experimental *q*_e_ and *C*_e_ values were highly variable, and no modelling could be performed (data not shown). However, the resulting *q*_e_ values were very low compared to PAC adsorption (e.g., the maximum *q*_e_ found was 530 µg/g for SMX), and thus, the adsorption onto the mixed liquor can be neglected for the pharmaceuticals under study [27]. However, the presence of additional suspended material (with a concentration of 6 g/L) could limit the ability of the pharmaceuticals to reach the PAC adsorption sites and, thus, physically reduce the adsorption of pharmaceuticals.

### 2.5. Influence of the Pre-Equilibrium Time between Pharmaceuticals and DOM on Adsorption

The influence on the interaction between DOM and the pharmaceuticals before the adsorption onto PAC was studied by using the HA solution. Humic acids are one of the most common DOM fractions found in wastewater [50], and they were chosen because of their commercial availability and ease of use in the laboratory. Because the objective was to study the interaction between DOM and the pharmaceuticals, DOC concentration does not have to be identical to the one found in the biological tank of the WWTP (4.7 mg/L). In fact, the experiments were performed with the highest possible DOC concentration, in order to produce the largest difference between DOM and the pharmaceutical concentration. The pre-contact time between HAs and OMPs was set at 24 h because it has already been tested as sufficient to evaluate the influence of the interaction between them [11].

The results of the adsorption isotherm parameters and correlation coefficients of the three pharmaceuticals with 24 h of pre-contact time are shown in Table 6, whereas the results of adsorption without a pre-equilibrium time are depicted in Table 5. Langmuir isotherm is the model that better fits the results in the HA solution in both conditions, and no statistical differences were found between them for the maximum adsorption capacity (*q*_m_) and Langmuir coefficient (*K*_L_). Regarding removal efficiencies (data not shown), no statistical differences were found between no pre-contact time and 24 h of pre-contact time with the HA solution, although a slight increment was observed for the condition without pre-contact time (3% for SMX, 7% for TMP and 8% for DCF). As explained earlier, the presence of HA in the solution had a neutral to positive effect for the three pharmaceuticals tested, which may be attributed to the high adsorption of the HAs and the interaction between the HA and the pollutants. However, the pre-contact time had no significant effect on the adsorption. The long shaking times of the adsorption experiments (24 h) were already sufficient to observe the potential beneficial effects of the presence of HA in the solution (e.g., formation of pharmaceutical–HA complexes, increased dispersion of the PAC), without the need for additional pre-contact time. In this way, in a previous study, it was found that the 24 h pre-contact time between DOM and various pharmaceuticals favored adsorption only at short contact times (i.e., 30 min) and had no effect once the equilibrium between the adsorbent and adsorbates was reached (i.e., 72 h) [11].

## 3. Materials and Methods

### 3.1. Adsorbent and Adsorbates

PAC (ACTISORBE 700, Brenntag S.p.A, Italy) was used for all the adsorption experiments. The PAC characteristics were supplied by the manufacturer as follows: iodine number 750 mg/g, methylene blue 12 mL, BET specific surface area 850 m^2^/g, bulk density 430 kg/m^3^, ash content 10%, humidity 5% and alkaline pH. The surface properties of the selected PAC are in agreement with the literature on adsorption of organic pollutants [18,51,52,53]. After its purchase, the PAC was not treated in order to emulate real conditions for which the adsorbent is directly added to the wastewater treatment line. 

The DCF, SMX and TMP properties are listed in Table 7. J Chem for Office (20.11.0, ChemAxon, https://www.chemaxon.com, accessed on 11 June 2021) was used for calculating the physicochemical properties (log*K*_ow_, molecular weight) and the ionization state (Figure 3). The calculation method for log*K*_ow_ is based on a modified version of the algorithm published by Viswanadhan et al. [54]. In this publication, the *K*_ow_ is the sum of the assigned values of the individual atomic contributions of a molecule. Molecular weight was based on the data published by IUPAC on the atomic weights of elements [55]. To calculate the ionization state, the software conducts a weighted sum of the net charges of the microspecies comprising the molecule as a function of the pH in aqueous solution. More information about the software functioning is available online. 

Diclofenac and sulfamethoxazole (≥98% TLC) were purchased from Sigma-Aldrich (St. Louis, MO, USA), and trimethoprim (≥98% TLC) was purchased from Acros Organics (Thermo Fisher Scientific Inc., Trenton, NJ, USA). To prepare the pharmaceutical solutions, exact amounts of the target compounds were weighed and added to the corresponding water matrix (Section 3.2). To ensure that the compounds were completely dissolved, a maximum of 1% of methanol was added, and the solutions were sonicated in an ultrasonic bath (Sonorex Digital 10P, Bandelin electronic, Berlin, Germany) for 5 min.

### 3.2. Water Matrices

Four different water matrices were used to prepare pharmaceutical solutions: ultra-pure water (Milli-Q), humic acid (HA) solution and effluent and mixed liquor from a WWTP. The preparation method of each water matrix is described below.

Milli-Q water was obtained from the Millipore Simplicity UV system (Millipore Corporation, Billerica, MA, USA).

Commercially available humic acids (CAS 1415-93-6, Sigma-Aldrich, St. Louis, MO, USA) were used to prepare the HA solution (50 mg/L), with a dissolved organic carbon (DOC) concentration of 29.35 mg/L. The solution was prepared following the method described by [48]. Briefly, to prepare a volume of 100 mL, 5 mL of 1M NH_4_OH were added to a 100 mL flask. Then, 0.005 g of HAs were weighed, and the Milli-Q water was added to a maximum of 85 mL. The pH of the solution was then adjusted to 5.34 with 1 M formic acid and prepared to the desired volume (100 mL).

The effluent and mixed liquor were collected from the permeate and the nitrification tank, respectively, of a full-scale MBR located in northern Italy, and frozen at −20 °C until their use. Both the MBR permeate and mixed liquor were autoclaved at 121 °C to reduce any potential biological activity and subsequently filtered through paper filters (Lab Expert, KEFO d.o.o, Croatia) to remove any particulate matter. Filters from the mixed liquor were air dried for 24 h and scrapped to obtain dry sludge. To ensure that all the glass beakers on which the adsorption experiments were conducted contained the same amount of mixed liquor suspended solids (MLSS), a certain amount (120 mg) of dry sludge was added to each glass baker. The resulting MLSS concentration in the mixed liquor was 6 g/L, a concentration commonly found in real WWTPs.

### 3.3. Batch Adsorption Experiments

Experiments were conducted in triplicate using 20 mL of pharmaceutical solutions in each glass beaker. The glass beakers were sealed with parafilm to avoid evaporation. All experiments were performed in triplicate using an incubator shaker at 150 rpm and a constant temperature of 25 °C (Innova 4080, New Brunswick Scientific, Edison, NJ, USA), which enabled continuous contact between the compounds and the activated carbon. To avoid photodegradation, all experiments were performed in darkness.

Preliminary experiments were conducted to determine the contact time necessary to reach the equilibrium between the PAC and the target pharmaceutical in ultra-pure water. Three different concentrations of target pollutants were tested (5, 15 and 25 mg/L). The PAC was agitated in the solutions for 10, 20, 30, 40 and 50 min and 1, 2, 4, 6, 12, 18 and 24 h at a constant temperature (25 °C). Two PAC concentrations (0.1 g/L and 1 g/L) were tested in each target compound individually, and 0.1 g/L of PAC was also tested in the mixture of the three pharmaceuticals. The results of the preliminary experiments determined 24 h to be sufficient time to reach the equilibrium for all three compounds and the mixture. Based on the results obtained, the sorption kinetics were determined. Kinetics studies were conducted by applying three different kinetics models: Lagergren pseudo-first-order [58] (1), pseudo-second-order (2) and intraparticle diffusion model (IPD) (3) [40].

(1) $$\frac{dq_{e}}{dt}=k_{1}\left(q_{e}-q_{t}\right)$$

(2) $$\frac{t}{q_{t}}=\frac{1}{k_{2}q_{e}^{2}}+\frac{1}{q_{e}}t$$

(3) $$q_{t}=k_{id}t^{1/2}+C$$

where *q*_e_ and *q*_t_ are the quantity of solute adsorbed onto the PAC surface (µg/g) at the equilibrium (*q*_e_) and at time t (*q*_t_); *k*_1_ (1/min), *k*_2_ (µg/g min) and *k*_id_ (µg/g·min^1/2^) are considered the Lagergren pseudo-first order, pseudo-second order and IPD rate constants, respectively; and intercept *C* provides information about the thickness of the boundary layer.

The batch sorption experiments were conducted in 20 mL of pharmaceutical solutions. For each water matrix, concentrations of pharmaceuticals ranging from 5 to 25 mg/L were tested to determine the sorption isotherms. PAC was added to the solutions at 0.1, 0.25, 0.5 and 1 g/L in each experiment and placed into agitation for 24 h. Equilibrium adsorption was studied by applying linear (4), Langmuir (5) and Freundlich (6) isotherm models to the experimental data,

(4) $$q_{e}=K_{d}C_{e}$$

(5) qe=KFCe1nqe=KFCe1n 

(6) $$\frac{1}{q_{e}}=\frac{1}{q_{m}}+\frac{1}{K_{L}q_{m}C_{e}}$$

where *q*_e_ is the amount of adsorbed compound per mass unit of adsorbent at the equilibrium (µg/g); *C*_e_ is the equilibrium concentration of the pharmaceutical (mg/mL); *K*_d_ is the distribution coefficient; *K*_F_ is the Freundlich adsorption constant ((µg/g) (mL/mg)^1/n^); 1/n is the heterogeneity constant; *q*_m_ is the equilibrium sorption capacity, that is, the maximum amount of OMP to be adsorbed by the activated carbon (µg/g); and *K*_L_ is the adsorption constant for Langmuir isotherms and is related to the sorption bonding energy (L/mg). Based on the four water matrices previously described, different experiments were conducted. Firstly, the pharmaceuticals were tested individually in each water matrix to compare the effect of the DOM (measured as DOC) in the adsorption process (ultra-pure water, humic acid solution, MBR permeate and mixed liquor). Secondly, sorption experiments were conducted in ultra-pure water with a mixture of the three target compounds (DCF, SMX and TMP) at the previously selected concentrations to evaluate the interaction and competition among the pharmaceuticals. Then, the HA solution was used to study the influence of a pre-equilibrium contact time between the DOM and the pharmaceuticals prior to the adsorption onto PAC. Pharmaceuticals were added to the HA solution 24 h before the addition of PAC to simulate their interactions in the sewer and inside the WWTP. Finally, mixed liquor experiments were performed with the addition of PAC and without PAC to assess the adsorption of the pharmaceuticals to the MLSS (i.e., added dried sludge).

### 3.4. HPLC Analysis

Prior to the quantitative analysis of the OMP concentration, glass beakers were decanted, and samples were centrifuged at 3500 rpm for 5 min (Hettich EBA 20, Westphalia, Germany) to subsequently be filtered with a 0.45 µm Nylon syringe filter (Filter-Bio, Nantong, China). Blank samples containing the corresponding water matrices were also included in the analysis to act as controls.

The residual pharmaceutical concentration was determined via high-performance liquid chromatography coupled to a photodiode array detection (HPLC-PDA) (Waters 2795 Separation Module and Waters 2996, Waters Corporation, Milford, MA, USA). A Kinetex C18 column was used (Phenomenex, 150 × 4.6 mm, 5 µm particle size, 100 Å pore size). The mobile phase contained eluent A, which was composed of 0.1% of formic acid in Milli-Q water, and solvent B, with 0.1% of formic acid in acetonitrile. The flow rate was 0.5 mL/min for all the experiments. The column temperature was 20 °C. The injection volume for each sample was 20 µL. Peak wavelengths are 276.9 nm for DCF, 269.8 for SMX and 270.8 nm for TMP. 

Isocratic methods were used to determine the concentrations of individual target pollutants. For DCF, the volume proportion of eluent A was 35%, and that of eluent B was 65%. For SMX, the proportions were 65% A and 35% B, whereas for TMP, the proportions were 85% A and 15% B. The total elution time was 10 min. The retention time was 6.5 min, 6 min and 5.6 min for DCF, SMX and TMP, respectively.

For the solution containing the mixture of pharmaceuticals, a method with gradient elution was developed. The total run time was 25 min, and the flow was kept constant at 0.5 mL/min. It started with a 1 min step gradient with 85% A and 15% B, which was then maintained as linear for another 5 min. Then, the flow was continued with a 1 min linear gradient with 65% A and 35% B, which was maintained for another 3 min; a 5 min gradient with 35% A and 65% B; and a step gradient of 0.1 min back to 85% A and 15% B, which was maintained for another 4.9 min. The retention time of each compound in the mixture was 6.2 min for TMP, 12.9 min for SMX and 20.2 min for DCF in the gradient elution method. 

## 4. Conclusions

The adsorption of three pharmaceuticals (namely DCF, SMX and TMP) onto PAC was studied through the use of kinetic and isotherm models in different water and wastewater matrices. Sorption of the tested pharmaceuticals was proven to be an overall fast process in ultra-pure water. Kinetics followed a pseudo-second order, suggesting that the sorption rate is governed by the number of available active sites. Additionally, the boundary layer effect seems to decrease the adsorption rate as compounds gradually reach the active sites at the equilibrium. Compared to individual solutions, the rate of the adsorption of the compounds in a mixture did not differ; however, a greater adsorption capacity of the PAC was observed in the individual solutions. 

Adsorption of pharmaceuticals onto the PAC surface is a complex process that greatly depends on physicochemical properties of the investigated compounds and on the matrix where it takes place. Charge, followed by hydrophobicity, determined the rate and the extent of the adsorption in all the tested matrices, with better results obtained via TMP (cationic compound), followed by DCF (anionic, hydrophobic) and SMX (anionic, hydrophilic). The effect of the water matrix varied from compound to compound. Humic acids appeared to positively affect the affinity for the adsorbent in DCF and SMX, presumably by forming pharmaceutical–HA complexes and by reducing the aggregation of PAC. Mixed liquor gave the lowest adsorption capacities of PAC, probably due to its complex nature and the presence of additional suspended solids. The adsorption isotherms also varied among water matrices. Only Langmuir isotherm explained adsorption in humic acid solution and Freundlich isotherm in the mixed liquor, whereas both isotherms fitted the results in ultra-pure water and MBR permeate very well. In this way, DOM and specifically HAs proved to be beneficial for the adsorption of the selected pharmaceuticals. However, the effects of the interaction of these elements prior to the addition of the adsorbent did not have an effect after long contact times (24h). In this way, future work should be focused on the understanding of the potential interactions between the organic components of the wastewater that may favor the adsorption of pharmaceuticals onto PAC.

## Figures and Tables

**Table 1:** Sorption kinetic parameters of DCF, SMX and TMP in ultra-pure water with 1 g/L and 0.1 g/L of added PAC. *C*_0_ indicates the initial concentration of the pharmaceutical, and *q*_e, exp._ indicates the values of *q*_e_ obtained experimentally.

Compound	PAC (g/L)	*C*_0_ (mg/L)	*q*_e, exp_. (µg/g)	Pseudo-First Order			Pseudo-Second Order		
				*q*_e, calc._ (µg/g)	*k*_1_ (1/min)	*R* ^2^	*q*_e, calc._ (µg/g)	*k*_2_ (g/µg·min)	*R* ^2^
DCF	1	5	4826	206	1.61 × 10^−4^	0.135	5000	4.00 × 10^−3^	1.000
	1	15	14,729	3185	2.07 × 10^−3^	0.806	14,286	6.13 × 10^−6^	1.000
	1	25	22,240	11,163	1.15 × 10^−3^	0.851	25,000	1.14 × 10^−6^	0.993
	0.1	5	31,442	127,321	6.91 × 10^−5^	0.743	33,333	1.13 × 10^−6^	0.999
	0.1	15	34,852	29,971	4.61 × 10^−4^	0.430	33,333	1.29 × 10^−6^	0.996
	0.1	25	34,869	229,192	4.61 × 10^−4^	0.877	33,333	6.92 × 10^−7^	0.995
SMX	1	5	4999	2085	5.07 × 10^−3^	0.987	5000	8.16 × 10^−6^	0.999
	1	15	9910	11,527	6.91 × 10^−4^	0.902	11,111	8.71 × 10^−7^	0.992
	1	25	11,549	23,206	4.61 × 10^−4^	0.877	14,286	1.88 × 10^−7^	0.979
	0.1	5	19,398	43,813	2.30 × 10^−4^	0.868	20,000	4.55 × 10^−7^	0.992
	0.1	15	26,490	138,038	9.21 × 10^−5^	0.784	25,000	7.41 × 10^−8^	0.996
	0.1	25	37,016	233,830	6.91 × 10^−5^	0.940	33,333	3.83 × 10^−8^	0.984
TMP	1	5	4992	82	2.07 × 10^−3^	0.598	5000	4.00 × 10^−7^	1.000
	1	15	14,933	606	1.84 × 10^−3^	0.543	14,286	4.90 × 10^−5^	1.000
	1	25	24,083	3151	1.15 × 10^−3^	0.657	25,000	8.00 × 10^−6^	1.000
	0.1	5	37,184	25,439	4.61 × 10^−4^	0.844	33,333	1.13 × 10^−6^	0.997
	0.1	15	33,416	126,765	6.91 × 10^−5^	0.561	33,333	1.5 × 10^−6^	0.999
	0.1	25	36,425	229,826	6.91 × 10^−5^	0.917	33,333	6.43 × 10^−7^	0.989

**Table 2:** Intraparticle diffusion model constants and correlation coefficients for DCF, SMX and TMP sorption at different initial concentrations (*C*_0_), together with the respective regression coefficients (*R*^2^). The PAC concentration used for the model is 1 g/L.

Compound	*C*_0_ (mg/L)	Intraparticle Diffusion								
		First Phase			Second Phase			Third Phase		
		*k*_p1_ (μg/g min^1/2^)	*C*_1_	*R* ^2^	*k*_p2_ (μg/g min^1/2^)	*C*_2_	*R* ^2^	*k*_p3_ (μg/g min^1/2^)	*C*_3_	*R* ^2^
DCF	5	0.402	93.03	0.921	0.078	95.45	1.000	−0.006	96.751	0.979
	15	15.657	129.49	0.996	2.322	242.66	0.999	−0.013	295.08	1.000
	25	26.047	109.34	0.976	11.310	192.21	0.962	0.029	443.70	1.000
SMX	5	2.596	50.37	0.985	1.926	54.95	0.962	0.074	97.16	1.000
	15	7.479	20.77	0.977	8.644	−8.99	1.000	0.187	191.09	1.000
	25	8.524	−23.18	0.947	14.061	−97.14	0.991	0.033	229.71	1.000
TMP	5	3.813	78.36	0.889	0.348	96.39	0.995	0.020	99.15	0.781
	15	12.055	211.25	0.958	0.958	285.80	0.998	0.072	296.07	0.938
	25	15.330	337.67	0.982	3.291	420.88	0.999	0.321	470.19	0.933

**Table 3:** Distribution coefficient (*K*_d_), Langmuir and Freundlich isotherm constants obtained in individual solutions of each pharmaceutical (DCF, SMX and TMP) and the mixture of the three pharmaceuticals in ultra-pure water. N.A. (not applicable) indicates that the parameters could not be obtained, as the residual concentration found in the liquid phase was too low to conduct the modelling.

		Linear Sorption		Langmuir Isotherm			Freundlich Sorption		
Compound	PAC Conc. (g/L)	*K*_d_ (mL/g)	*R* ^2^	*q*_m_ (µg/g)	*K*_L_ (L/mg)	*R* ^2^	1/n	*K*_F_ (mg/g) (mL/mg)^1/n^	*R* ^2^
Individual solutions									
DCF	0.1	1777.9	0.895	33,333	0.300	0.963	0.281	12,673.9	0.925
	0.25	1949.2	0.836	33,333	0.429	0.979	0.215	14,368.6	0.953
	0.5	2980.6	0.783	25,000	2.000	0.978	0.271	14,099.6	0.991
	1	7167.1	0.855	20,000	5.000	0.946	0.574	10,802.1	0.999
SMX	0.1	1896.0	0.960	50,000	0.100	0.915	0.439	8918.7	0.959
	0.25	1634.0	0.947	33,333	0.150	0.936	0.392	7972.1	0.967
	0.5	1756.3	0.902	25,000	0.444	0.956	0.380	7667.1	0.985
	1	1417.6	0.937	16,667	0.300	0.912	0.520	3947.0	0.990
TMP	0.1	2618.9	0.833	50,000	0.400	0.951	0.178	23,576.4	0.801
	0.25	3712.3	0.820	50,000	0.667	0.972	0.249	21,407.6	0.961
	0.5	5939.4	0.852	33,333	1.500	0.967	0.393	16,565.9	0.998
	1	19,820.0	0.910	25,000	4.444	0.939	N.A.	N.A.	N.A.
Mixture									
DCF	0.1	1806.2	0.852	33,333	0.375	0.987	0.203	16,008.9	0.955
	0.25	1063.6	0.763	16,667	1.000	1.000	0.124	11,356.0	0.900
	0.5	1348.8	0.785	16,667	1.000	0.995	0.212	9531.0	0.962
	1	1390.1	0.707	12,500	1.000	0.996	0.125	9464.5	0.823
SMX	0.1	423.03	0.935	50,000	0.010	0.031	0.587	1222.7	0.423
	0.25	385.32	0.965	14,286	0.054	0.924	0.670	1012.2	0.950
	0.5	280.47	0.976	10,000	0.053	0.869	0.709	629.0	0.998
	1	162.16	0.868	3333	0.375	0.968	0.137	1783.6	0.652
TMP	0.1	2442.7	0.901	50,000	0.200	0.832	0.257	17,243.7	0.597
	0.25	2036.5	0.733	25,000	2.000	0.999	0.128	19,171.9	0.964
	0.5	2716.2	0.730	25,000	2.000	0.999	0.151	17,870.4	0.955
	1	5636.6	0.843	25,000	2.000	0.995	0.239	14,485.5	1.000

**Table 4:** Sorption kinetic parameters for the mixture of DCF, SMX and TMP in ultra-pure water with 0.1 g/L of added PAC.

Compound	*C*_0_ (mg/L)	*q*_e, exp._ (µg/g)	Pseudo-First Order			Pseudo-Second Order		
			*q*_e, calc._ (µg/g)	*k*_1_ (1/min)	*R* ^2^	*q*_e, calc._ (µg/g)	*k*_2_ (g/µg·min)	*R* ^2^
DCF	5	18,467	136,395	9.21 × 10^−5^	0.878	16,667	1.33 × 10^−6^	0.991
	15	28,362	40,272	1.84 × 10^−4^	0.851	33,333	4.09 × 10^−7^	0.993
	25	15,957	242,493	2.30 × 10^−5^	0.387	16,667	1.2 × 10^−6^	0.990
SMX	5	5716	48,865	6.909 × 10^−5^	0.801	10,000	1.81 × 10^−7^	0.890
	15	4742	147,809	1.382 × 10^−5^	0.633	5000	2.72 × 10^−6^	0.991
	25	35,771	237,684	6.909 × 10^−5^	0.740	33,333	2.81 × 10^−7^	0.997
TMP	5	25,531	32,464	2.30 × 10^−4^	0.820	25,000	1.45 × 10^−6^	0.999
	15	25,310	134,122	4.61 × 10^−5^	0.435	25,000	1.23 × 10^−6^	0.990
	25	25,948	239,111	4.61 × 10^−5^	0.874	25,000	5.71 × 10^−7^	0.941

**Table 5:** Distribution coefficient (*K*_d_), Langmuir and Freundlich isotherm constants in different water matrices (humic acid solution, MBR permeate and mixed liquor). Results for humic acid solutions were considered without pre-contact time between the HAs and the pharmaceuticals. N.A. (not applicable) indicates that the parameters could not be obtained, as the residual concentration found in the liquid phase was very low to conduct the modelling.

		Linear Sorption		Langmuir Isotherm			Freundlich Sorption		
Compound	PAC Conc. (g/L)	*K*_d_ (mL/g)	*R^2^*	*q*_m_ (µg/g)	*K*_L_ (L/mg)	*R* ^2^	1/n	*K*_F_ (mg/g) (mL/mg)^1/n^	*R* ^2^
Humic acid solution									
DCF	0.1	4521.6	0.941	100,000	0.125	0.908	0.4568	18,012.1	0.929
	0.25	4802.4	0.783	50,000	1.000	0.994	0.2799	24,760.4	0.896
	0.5	4600.6	0.768	33,333	1.500	0.984	0.2000	20,607.3	0.781
	1	12,308.0	0.718	100,000	1.429	0.994	N.A.	N.A.	N.A.
SMX	0.1	2856.7	0.878	50,000	0.250	0.919	0.2630	20,426.7	0.863
	0.25	3957.7	0.792	50,000	1.000	0.983	0.1408	29,673.2	0.651
	0.5	6994.4	0.801	33,333	3.000	0.983	0.2731	22,606.7	0.807
	1	11,372.0	0.763	25,000	5.000	0.991	N.A.	N.A.	N.A.
TMP	0.1	2287.9	0.860	50,000	0.286	0.976	0.2116	19,150.8	0.900
	0.25	2600.1	0.791	33,333	0.750	0.992	0.1891	19,424.7	0.958
	0.5	3824.5	0.720	33,333	3.000	0.994	0.1960	19,358.8	0.998
	1	31,430.0	0.740	25,000	10.000	0.998	N.A.	N.A.	N.A.
MBR permeate									
DCF	0.1	1553.7	0.880	33,333	0.150	0.978	0.4160	8206.6	0.865
	0.25	1785.4	0.802	25,000	0.667	0.989	0.2066	14,004.0	0.925
	0.5	3273.4	0.776	50,000	1.000	0.985	0.2785	14,831.4	0.997
	1	12,011.0	0.734	25,000	1.000	0.995	N.A.	N.A.	N.A.
SMX	0.1	1642.8	0.999	1,000,000	0.002	0.028	0.9527	1843.1	0.988
	0.25	1349.2	0.962	33,333	0.100	0.924	0.4976	5154.4	0.978
	0.5	1874.2	0.870	25,000	0.444	0.993	0.2650	10,690.4	0.932
	1	3009.7	0.837	20,000	1.000	0.999	0.2310	11,178.0	0.996
TMP	0.1	9370.2	0.875	250,000	0.057	0.225	0.8102	15,532.7	0.647
	0.25	6616.8	0.690	50,000	1.000	0.974	0.2822	31,351.0	0.754
	0.5	8417.5	0.535	50,000	1.000	0.937	N.A.	N.A.	N.A.
	1	N.A.	N.A.	N.A.	N.A.	N.A.	N.A.	N.A.	N.A.
Mixed liquor									
DCF	0.1	827.9	0.993	−25,000	−0.019	0.466	1.3563	299.7	0.957
	0.25	766.7	0.963	−10,000	−0.033	0.407	1.6076	148.1	0.903
	0.5	235.9	0.995	50,000	0.005	0.038	0.9064	296.5	0.952
	1	234.1	0.998	33,333	0.008	0.268	0.8891	312.6	0.979
SMX	0.1	431.5	0.965	−3333	−0.048	0.907	1.8270	55.0	1.000
	0.25	233.2	0.990	−33,333	−0.006	0.085	1.0983	186.3	0.954
	0.5	84.0	0.892	2000	0.172	0.873	0.4847	384.3	0.707
	1	109.4	0.858	2500	0.118	0.552	0.6615	300.6	0.594
TMP	0.1	3988.7	0.976	1,250,000	0.004	0.015	1.0055	4011.8	0.939
	0.25	1785.7	0.995	125,000	0.020	0.538	0.8440	2659.3	0.980
	0.5	1002.7	0.960	33,333	0.060	0.986	0.6493	2484.4	1.000
	1	822.7	0.868	14,286	0.233	0.996	0.4847	2980.3	0.967

**Table 6:** Distribution coefficient (*K*_d_) and Langmuir and Freundlich isotherm parameters, together with the corresponding regression coefficients (*R*^2^) for the adsorption DCF, SMX and TMP onto PAC in a humic acid solution with 24 h pre-contact time between the pharmaceuticals and the humic acids. Not applicable (N.A.) indicates that the parameters could not be obtained, as the residual concentration found in the liquid phase was too low to conduct the modelling.

		Linear Isotherm		Langmuir Isotherm			Freundlich Isotherm		
Compound	PAC Conc. (g/L)	*K*_d_ (mL/g)	*R^2^*	*q*_m_ (µg/g)	*K*_L_ (L/mg)	*R^2^*	1/n	*K*_F_ (mg/g) (mL/mg)^1/n^	*R^2^*
DCF	0.1	2931.5	0.8482	50,000	0.400	0.983	0.2194	23,435.3	0.951
	0.25	3196.9	0.8713	50,000	0.333	0.980	0.3342	15,739.0	0.988
	0.5	2932.1	0.7000	25,000	2.000	0.999	0.0962	20,854.0	0.959
	1	2403.6	0.6190	16,667	6.000	0.990	0.0825	13,418.6	0.614
SMX	0.1	2378.1	0.8972	50,000	0.250	0.985	0.2619	18,030.1	0.953
	0.25	3434.0	0.8685	50,000	0.500	0.981	0.3040	18,300.8	0.970
	0.5	5491.8	0.8108	33,333	3.000	0.988	0.1885	23,086.4	0.948
	1	7530.7	0.7846	20,000	6.250	0.988	N.A.	N.A.	N.A.
TMP	0.1	2340.0	0.8716	50,000	0.250	0.989	0.2932	16,000.9	0.9487
	0.25	2567.3	0.7911	33,333	0.750	0.991	0.2272	17,667.8	0.9735
	0.5	3896.4	0.7282	33,333	3.000	0.996	0.1366	22,092.6	0.9872
	1	N.A.	N.A.	N.A.	N.A.	N.A.	N.A.	N.A.	N.A.

**Table 7:** Physicochemical properties of the selected pharmaceuticals. J Chem for Office (20.11.0, ChemAxon, https://www.chemaxon.com, accessed on 11 June 2021) was used for calculating the physicochemical properties (molecular weight and log*K*_ow_). Values for p*K*_a1_ and p*K*_a2_ were obtained from the literature [56,57].

Compound	Molecular Formula	Molecular Weight (g/mol)	log*K*_ow_^1^	p*K*_a1_	p*K*_a2_
Diclofenac	C_14_H_10_Cl_2_NNaO_2_	318.13	4.26	4.21 ^2^	
Sulfamethoxazole	C_10_H_11_N_3_O_3_S	253.28	0.79	1.83 ^2^	5.57 ^2^
Trimethoprim	C_14_H_18_N_4_O_3_	290.32	1.28	7.10 ± 0.02 ^3^	

^1^ Octanol–water partition coefficient. ^2^ Obtained from [56]. ^3^ Obtained from [57].

## Data Availability

Not applicable.

## Sample Availability

Samples of the compounds are not available from the authors.
